# Prevalence and treatment of heart failure in Swedish nursing homes

**DOI:** 10.1186/1471-2318-13-118

**Published:** 2013-11-05

**Authors:** Beata Borgström Bolmsjö, Sigvard Mölstad, Carl Johan Östgren, Patrik Midlöv

**Affiliations:** 1 Department of Clinical Sciences in Malmö, Center for Primary Health Care Research, Lund University, Malmö, Sweden; 2 Department of Medical and Health Sciences, Primary Care, Linköping University, Linköping, Sweden

## Abstract

**Background:**

Since the burden of care for elderly patients with heart failure (HF) can be decreased by therapeutic measures, it is important that such patients are identified correctly. This study explores the prevalence of HF in nursing homes in Sweden, with special consideration of the risk of failure to diagnose HF in the study population. A second aim is to explore medication and the adherence to guidelines for the treatment of HF.

**Methods:**

429 patients from 11 nursing homes were included during 2008–2011. Information about diagnoses and medications from patient records, blood samples, questionnaire responses and blood pressure measurements were collected. The baseline characteristics of the patients, their medications and one-year mortality were identified and then compared regarding HF diagnosis and B-type natriuretic peptide (BNP) levels. A BNP level of >100 ng/L was used to identify potential cases of HF.

**Results:**

The point prevalence of HF diagnosis in the medical records in the study population was 15.4%. With the recommended cut-off value for BNP, up to 196 subjects in the study population (45.7%) qualified for further screening of potential HF.

The subjects in the HF and non-HF groups were similar with the exception of mean age, BNP levels and Mini Mental State Examination results which were higher in the HF group, and the eGFR and blood pressure, which were lower when HF. The subjects with higher BNP values were older and had lower eGFR, Hb, diastolic blood pressure and BMI. The subjects with HF diagnoses were in many cases not treated according to the guidelines. Loop diuretics were often used without concomitant ACE inhibitors or angiotensin receptor blockers. The subjects without HF diagnoses in the medical records at inclusion but with BNP values >100 ng/L had less appropriate HF medication. The one-year mortality was 52.9% in the population with HF.

**Conclusions:**

Our study suggests that the estimated prevalence of HF in nursing homes in Sweden would increase if BNP measurements were used to select patients for further examinations. The pharmacological treatment of HF varied substantially, as did adherence to guidelines.

## Background

The prevalence of HF varies around the world, because of different panorama of diseases, survival after myocardial infarctions, incidence of valvular heart diseases and the rate of prevention measures. Epidemiologic studies from the developing countries are however lacking. The general prevalence of HF is estimated to be around 1-2% in the western world [1]. The prevalence of HF rises with age and persons younger than 50 years are hardly ever found to have HF. In the US, the prevalence of HF is about 0.7% in the 45–54 years of age and 8.4% for those aged 75 years and older [2].

The prevalence of HF in the elderly is hard to estimate accurately because of both the atypical presentation of HF in elderly patients and the lack of large studies in this population [3,4]. Studies on elderly persons in Europe show a prevalence of HF from 23% in nursing home residents in the Netherlands [5] up to almost 50% in 87–89 year olds in the UK [3]. In Sweden a population-based study from 2001 show a HF prevalence of 6.7% in men and women at the age of 75 [6]. There is no updated study on the prevalence of HF in the elderly population over 75 years of age in Sweden.

The proportion of elderly is growing in the western world. The part of the population aged 65 and over in Sweden is around 20% corresponding to 1.8 million people [7]. The proportion older elderly i.e. over 80 years of age is continually rising and is presently 5.2% [7] and expected to increase to one tenth of the population in 2050 [8].

The first evaluation of HF should be based on a well-conducted anamnesis followed by a thorough physical examination and appropriate laboratory tests [4]. Symptoms such as fatigue, confusion, memory deficit, irritability, anorexia and a gradual reduction in level of activity are common manifestations of HF in individuals aged above 80 years [4]. Dementia is common in nursing home populations, and a careful medical history, which is crucial for the diagnosis of HF, may be difficult in residents with cognitive impairment. It was therefore suggested that the diagnosis of HF in this group of patients is inadequate [9]. The great variability in the detection and interpretation of signs and symptoms by physicians is associated with low sensitivity and specificity in the clinical diagnosis of HF in the elderly [10]. In nursing homes, adherence to guidelines for diagnostic investigations and treatment is notoriously lax [11].

The recommended treatment of HF in the elderly is similar to that of younger cohorts with respect to renal function and the risk of polypharmacy. Not many clinical trials have been conducted on elderly patients. However, the benefit of adherence to treatment guidelines for HF is evident as it reduces the morbidity and mortality in elderly patients, as well as in younger patients [12].

β-type natriuretic peptide (BNP) is a hormone produced mainly by ventricular cardiomyocytes. Its secretion is associated with stretching of myocardial fibres. Quantitative analysis of plasma concentrations of BNP is useful to help confirming the diagnosis, provide a prognosis and guide treatment in patients with HF [4,13]. BNP cannot be used to replace cardiac imaging to confirm a final diagnosis [10]. The use of BNP as a prognostic marker in patients with HF has proved to be essential for identifying frail elderly patients at risk of events as hospitalization [11]. There are several different confounders involved in the interpretation of BNP as the BNP levels are shown to rise with age, female sex and impaired renal function [14]. The studies of the use of BNP for further diagnosis of HF in an elderly population are limited.

In Sweden 5.2% of the persons aged 65 and over live permanently in nursing homes [15]. Elderly living in nursing homes in Sweden are frail and have several co-morbidities along with polypharmacy [16]. These individuals are taken care of by a permanent nursing home physician who usually visits the nursing home once weekly. Hence, the elderly do not have to go to the hospital for examinations as the doctor comes to them. The patients only go to the hospital when acute illness or traumas with need of hospital care. It is therefore important for the physician to carefully select the patients in need of further examinations as echocardiography. If BNP measures could be used, one would be able to minimize unnecessary examinations for this frail population.

The aim of this study is to explore the prevalence of HF in nursing homes in Sweden, with special consideration of the risk of neglected HF diagnoses, by use of BNP measurements. A second aim is to explore medication and the adherence to guidelines for the treatment of HF.

## Methods

### Study population

The Study of Health and Drugs in the Elderly (SHADES) is a longitudinal cohort study of elderly people living in 11 different nursing homes in three cities in the southern part of Sweden (Linköping, Jönköping and Eslöv). The aim of the SHADES study is to map the mortality, morbidity and pharmaceutical treatment of elderly residents in nursing homes and to use the data collected to improve health care in the elderly [17]. One of the major objectives for the SHADES study is to further explore HF in a nursing home population.

All residents of the 11 selected nursing homes were invited to join the study and when included residents moved or died, the next person moving in to the nursing home was asked to participate. During 2008–2011, 429 patients were included in the SHADES study. The mean age of the participants was 85.0 years with a range between 65 and 101 years. Patients who lived at the nursing home temporarily for short-term rehabilitation or palliative care were excluded. Persons with language difficulties and persons under the age of 65 were also excluded.

**Figure 1:**
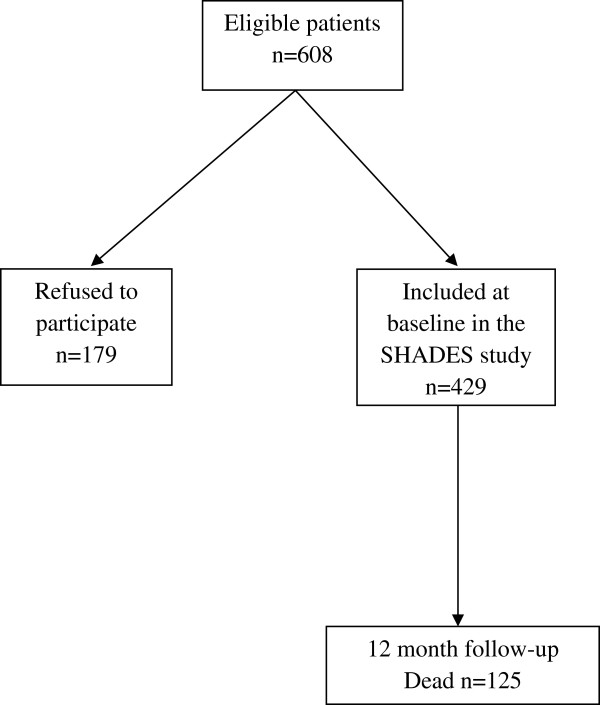
Flow chart of the patients in the SHADES study.

The subjects included, and those subjects who were excluded, moved or died, are presented in Figure 1. There are no regional differences regarding nursing homes within Sweden.

### Methods of investigation

Participants were examined at baseline of the study by specially trained nurses who also collected data from patient records for diagnoses and current medical treatment. In this study we followed the patients during one year after inclusion to get one-year mortality data.

Diagnoses collected from the patients records were coded according the Swedish version of the 10^th^ version of the International Classification of Diseases (ICD-10) [18]. The subjects with the ICD-10 code I50 in their patient record at inclusion were selected as patients with HF diagnoses.

The in-person testing of participants included measurement of pulse, blood pressure, weight and height, and questionnaires. The in-person testing was performed by the study nurses with assistance of the staff at the nursing home. Blood pressure was measured three times with one minute apart, in the right arm with the respondent sitting. The mean value of these three measurements was used for blood pressure analyses.

To measure cognitive function, the Mini Mental State Examination (MMSE) was used [19]. The MMSE consists of 21 questions that measure orientation, memory, naming, constructional ability and attention. The scores range from 0 to 30, with a score of 23 or lower indicating cognitive dysfunction.

Blood samples were drawn according to a standard procedure and were stored at -70°C in a freezer. All blood samples were analysed at the hospital in Jönköping by high-pressure liquid chromatography.

For BNP measurements, a cut off value of 100 ng/L is suggested to have a satisfactory negative predictive value and satisfactory sensitivity for determining the need for further investigation of HF in primary health care [20].

A chemiluminescent micro particle assay (CMIA: Architect i2000 BNP assay, Abbot Laboratories, Abbot Park, IL, USA) was used for the quantitative determination of BNP concentrations in EDTA plasma. The results from the laboratory analyses are presented in ng/L, with the method range of 10–5000 ng/L, <10 ng/L and >5000 ng/L, and the reference value set to <100 ng/L [21].

We used the formula for assessing renal function by estimating glomerular filtration rate (GFR) according to recently updated Swedish guidelines [22]. The estimated GFR (eGFR) was defined as the average of (1) the GFR estimated from creatinine based on the revised equations for estimating GFR from the Lund-Malmo Study cohort [23] and (2) the GFR estimated from cystatin C [24].

The HF prevalence in the cohort was calculated as the point prevalence where the amount of patients with the ICD-10 diagnosis HF in their patient record at the time for inclusion was divided by the total amount of subjects at inclusion.

The patients’ medical treatment was reviewed with respect to current Swedish [25] and European [26] guidelines. ACE inhibitors are recommended in the first instance (angiotensin receptor blockers (ARBs) are recommended in cases of intolerance to ACE inhibitors), preferably combined with Beta-blockers, followed by a mineralocorticoid/aldosterone receptor antagonist (e.g. spironolactone) when symptoms persist. Digoxin is additionally recommended to control the ventricular rate in patients with an inadequate response to Beta-blocker treatment. Diuretics are additionally recommended in patients with signs and symptoms of congestion.

One-year mortality was calculated by number of deaths over number of person-year lived over one year. The subjects who joined the study at the latest period of inclusion were followed at least 12 months after inclusion to get adequate mortality data. For mortality comparisons the population was divided into age strata, gender and HF diagnosis.

### Ethics

The study protocol was approved by the Regional Ethics Review Board at Linköping University (date: October 18, 2007; case number M150-07). Informed consent was obtained from all participants. If the patient could not understand the information and give informed consent this was obtained from next of kin.

### Statistical analysis

The data collected in the study were analysed using the SPSS Statistics 20 (SPSS, Inc. Chicago, IL). Differences between groups were tested using Student’s *T*-test and the Mann–Whitney *U* test for continuous variables and the Chi-square test for discrete variables.

For calculating differences between several groups the one-way ANOVA test was done for continuous variables and Chi square test for discrete variables, using the Bonferroni correction for mass significance. With the Bonferroni calculation for mass significance when six comparisons were made, the p value for significant differences was corrected from p < 0.05 to p <0.0083.

Binary logistic regression analysis with the Enter method was made for observing differences between the groups with HF diagnosis and the group with no HF diagnosis but with BNP > 100 ng/L. The goodness-of-fit of the regression model was tested with the Hosmer and Lemeshow test, and with Nagelkerke R^2^.

## Results

### Baseline characteristics

The point prevalence of diagnoses of HF in the patient records at the time for inclusion was 15.4% in our study population. The characteristics of the subjects in the group with HF diagnoses, compared to the subjects without HF diagnoses, are shown in Table 1. The subjects in the two groups were quite similar. The population with HF diagnoses was older than the population without HF diagnoses (86.8 compared to 84.7 years p = 0.024). The BNP values were higher and eGFR as well as blood pressure were lower in the group with HF diagnoses. The group with diagnosed HF had significantly higher MMSE scores compared to the group without diagnosed HF. The prevalence of hypertension was similar in both groups.

**Table 1:** Baseline characteristics of the population with HF diagnoses compared to the population with no diagnosis of HF

**Parameter**	**All**	**No HF diagnosis**	**HF diagnosis**	**p value**
	**(n = 429)**	**(n = 363)**	**(n = 66)**	**HF vs. no HF**
Point prevalence of HF diagnosis (%)	15.4	-	100	
BNP range (ng/L)	10-4200	10-1487	30-4200	*p = 0.002*
	(n = 402)	(n = 342)	(n = 60)	
	Median 103.5	Median 89.5	Median 210.5	*p < 0.001*
BNP (ng/L) mean ± SD	181.1 ± 299.6	143.2 ± 178.6	397.4 ± 608.1	*p = 0.002*
Male sex n (%)	124 (28.9)	104 (28.7)	20 (30.3)	p = 0.79
Age (years) mean ± SD	85.0 ± 7 (n = 429)	84.7 ± 7.1 (n = 363)	86.8 ± 5.6 (n = 66)	*p = 0.024*
P-glucose (mmol/L) mean ± SD	5.6 ± 1.9 (n = 390)	5.6 ± 1.9 (n = 332)	5.6 ± 1.7 (n = 58)	p = 0.81
hs-CRP (mg/L) mean ± SD	3.8 ± 2.6 (n = 266)	3.8 ± 2.6 (n = 232)	4.2 ± 2.8 (n = 34)	p = 0.32
TSH (mIE/L) mean ± SD	2.0 ± 2.5 (n = 406)	2.1 ±2.6 (n = 343)	1.8 ± 1.4 (n = 63)	p = 0.40
Hb (g/L) men mean ± SD	128.5 ± 15.4 (n = 115)	129.4 ± 14.3 (n = 97)	123.8 ± 20.1 (n = 18)	p = 0.16
Hb (g/L) women mean ± SD	124.5 ± 13.6 (n = 276)	124.9 ± 13.4 (n = 236)	121.9 ± 14.2 (n = 40)	p = 0.19
eGFR (ml/min/1.73 m^2^) mean ± SD	44.4 ± 16.6 (n = 406)	45.9 ± 15.5 (n = 344)	35.9 ± 16.4 (n = 62)	*p < 0.001*
BMI (kg/m^2^) mean ± SD	24.9 ± 5.6 (n = 420)	24.8 ± 4.9 (n = 355)	25.5 ± 5.8 (n = 65)	p = 0.30
MMSE result mean ± SD	17.1 ± 6.5 (n = 359)	16.8 ± 6.4 (n = 299)	18.6 ± 6.7 (n = 60)	*p = 0.061*
Systolic blood pressure (mmHg) mean ± SD	133.5 ± 23.4 (n = 407)	134.5 ± 23.5 (n = 344)	127.9 ± 21.8 (n = 63)	*p = 0.038*
Diastolic blood pressure (mmHg) mean ± SD	72.2 ± 11.7 (n = 407)	72.7 ± 11.7 (n = 345)	69.4 ± 11.6 (n = 62)	*p = 0.040*
Hypertension n (%) diagnosis	128 (29.8)	108 (29.8)	20 (30.3)	p = 0.93

### BNP

The study population was divided into quartiles based on BNP level and the characteristics of the subjects in the four groups are presented in Table 2. Only 32% of the subjects in the fourth BNP quartile had been diagnosed with HF. The MMSE results did not differ between the different quartiles. The prevalence of hypertension did not differ between the quartiles. The baseline characteristics between the quartiles differed in Hb, blood pressure, eGFR, thyroid-stimulating hormone (TSH), and body mass index (BMI), as well as for age (p < 0.05) (Table 2).

**Table 2:** Baseline characteristics of the study population, divided into quartiles based on BNP

**BNP quartile**	**1**	**2**	**3**	**4**	**p value**
BNP range (ng/L)	10-51	52-102	103-191	192-4200	
	(n = 100)	(n = 101)	(n = 101)	(n = 100)	
BNP (ng/L) mean ± SD	31.2 ± 10.9	74.8 ± 14.4	148.7 ± 24.7	471.2 ± 492.3	*p < 0.001*
	(n = 100)	(n = 101)	(n = 101)	(n = 100)	*b, c, e, f*
Male sex (%)	35 (n = 35)	26.7 (n = 27)	29.7 (n = 30)	26 (n = 26)	p = 0.49
Age (years) mean ± SD	81.1 ± 7.3	84.8 ± 6.5	87.1 ± 5.5	87.2 ± 6.1	*p < 0.001*
	(n = 100)	(n = 101)	(n = 101)	(n = 100)	*a, b, c*
P-glucose (mmol/L) mean ± SD	6.0 ± 2,5	5.5 ± 1.5	5.6 ± 1.6	5.5 ± 1.7	p = 0.21
	(n = 96)	(n = 95)	(n = 99)	(n = 95)	
hs CRP (mg/L) mean ± SD	3.7 ± 2.8	4.0 ± 2.7	3.5 ± 2.5	4.2 ± 2.6	p = 0.22
	(n = 68)	(n = 67)	(n = 68)	(n = 59)	
TSH (mIE/L) mean ± SD	2.8 ± 3.8	1.7 ± 1.8	1.9 ± 2.2	1.7 ± 1.1	*p = 0.003*
	(n = 100)	(n = 99)	(n = 101)	(n = 100)	*a, b, c*
BMI (kg/m^2^) mean ± SD	26.8 ± 5	24.8 ± 4.6	24.0 ± 4.4	23.9 ± 4.8	*p < 0.001*
	(n = 98)	(n = 98)	(n = 101)	(n = 98)	*a, b, c*
eGFR (ml/min/1.73 m^2^) mean ± SD	51.1 ± 15.2	46.1 ± 14.8	41.0 ± 15.1	39.5 ± 16.3	*p < 0.001*
	(n = 100)	(n = 101)	(n = 99)	(n = 99)	*b, c, e*
Hb in men (g/L) mean ± SDHb in women (g/L) mean ± SD	135.3 ± 13.7	128.7 ± 14.1	125.7 ± 13.9	123.0 ± 17.4	*p = 0.010*
	(n = 35)	(n = 25)	(n = 29)	(n = 25)	*c*
	125.2 ± 12.7	125.7 ± 13.0	124.2 ±13.8	122.6 ±14.7	p = 0.54
	(n = 63)	(n = 72)	(n = 68)	(n = 70)	
Systolic blood pressure (mmHg) mean ± SD	134.8 ± 19.6	132.8 ± 20.7	136.0 ± 26.9	131.3 ± 25.8	p = 0.47
	(n = 97)	(n = 97)	(n = 99)	(n = 95)	
Diastolic blood pressure (mmHg) mean ± SD	74.0 ± 10.1	73.5 ± 11,5	70.2 ± 12.0	70.7 ± 12.5	*p = 0.045*
	(n = 97)	(n = 97)	(n = 99)	(n = 95)	
HF diagnosis n (%)	7 (7)	4 (4)	17 (16.8)	32 (32)	*p < 0.001*
					*c, d, e*
Hypertension diagnosis n (%)	31 (31)	27(26.7)	34 (33.7)	33 (33)	p = 0.71
MMSE result mean ± SD	17.0 ± 6.6	17.0 ± 6.6	17.2 ± 7.0	17.2 ± 5.8	p = 0.89
	(n = 80)	(n = 87)	(n = 92)	(n = 88)	

Significant difference between BNP quartiles 1–4, is presented as:

1 and 2: a.

1 and 3: b.

1 and 4: c.

2 and 3: d.

2 and 4: e.

3 and 4: f.

Based on the recommended BNP cut-off for HF of >100 ng/L, 196 subjects in the study population may have had HF, while only 66 had the diagnosis in the medical charts. In the group with no HF diagnosis at the time for inclusion the mean BNP level was 143.2 ng/L, and 154 subjects in this group should have been further examined for potential HF.

### High BNP values but no HF

The study population was divided into those with HF diagnosis in the medical record at inclusion and those with no HF diagnosis but with BNP values >100 ng/L. This is presented in Table 3.

**Table 3:** Logistic regression analysis concerning age, gender, renal function and medications in relation to the population with HF diagnoses and the population with no diagnosis of HF and BNP > 100 ng/L

**Variable**	**HF diagnoses (n = 66)**	**No HF diagnoses and BNP > 100 ng/L (n = 154)**	**p-value**	**OR**	**CI 95% for OR**
Age (years)	86.8 ± 5.6 (n = 66)	87.0 ± 6.0 (n = 66)	0.82	1.02	0.94-1.08
Male sex n (%)	20 (30.3)	44 (28.6)	0.91	0.96	0.44-2.10
eGFR (ml/min/1.73 m^2^)	35.9 ± 16.4 (n = 62)	42.1 ± 15.4 (n = 152)	0.27	0.99	0.96-1.01
ACE inhibitors/ARBs	33 (50)	23 (14.9)	*<0.001*	11.27	3.14-40.41
Beta-blockers	39 (59.1)	70 (45.5)	0.99	1.00	0.48-2.08
Spironolactone	10 (15.2)	8 (5.2)	*0.04*	3.55	1.08-11.68
Digoxin	16 (24.2)	14 (9.1)	0.31	1.66	0.62-4.43
Furosemide/loop diuretics	50 (75.8)	50 (32.5)	*<0.001*	5.97	2.39-14.92
Loop diuretics **with** simultaneous ACE inhibitor/ARB treatment	24 (36.4)	35 (22.7)	0.08	0.25	0.05-1.16

Nagelkerke R^2^ 0.34.

Hosmer and Lemeshow test p-value 0.74.

### Pharmaceutical treatment

The patients with diagnosed HF used more drugs than the subjects without HF diagnoses but with BNP > 100 ng/L (8.5 vs. 7.0 medications on average, p < 0.001). The most commonly used medications were loop diuretics followed by Beta-blockers in the HF group (used in 75.8% and 59.1% of the subjects respectively) and Beta-blockers followed by loop diuretics in the non-HF group with BNP > 100 ng/L (used in 45.5% and 32.5% of the subjects respectively). Treatment with ACE inhibitors/ARBs were used in 50% of the subjects with HF diagnosis and in 14.9% in subjects without HF diagnosis but with BNP > 100 ng/L. Subjects with diagnosis of HF were more likely to be treated with ACE inhibitors/ARBs, Spironolactone and loop diuretics (Table 3). For the treatment with Digoxin and Beta-blockers the groups were similar. The subjects with high BNP values had more medications compared to the subjects with lower BNP values (the average number of medications per subject was 7.7 for the fourth BNP quartile and 6.0 in the first BNP quartile). The subjects in the fourth quartile were more likely to be treated with Beta-blockers, Digoxin and loop diuretics (Table 4). The groups did not differ in the treatment with ACE inhibitors/ARBs or Spironolactone.

**Table 4:** HF medication in the study population, divided into quartiles based on BNP

**BNP quartile**	**1**	**2**	**3**	**4**	**p value**
**Range (ng/L)**	**10-51**	**52-102**	**103-191**	**192-4200**	
	**(n = 100)**	**(n = 101)**	**(n = 101)**	**(n = 100)**	
ACE inhibitors/ARBs n (%)	14 (14)	20 (19.8)	20 (19.8)	28 (28)	0.11
Beta-blockers n (%)	14 (14)	31 (30.7)	45 (44.6)	54 (54)	*<0.001*
					*a, b, c, e*
Spironolactone n (%)	9 (9)	4 (4)	7 (6.9)	7 (7)	0.56
Digoxin n (%)	2 (2)	1 (1)	13 (12.9)	14 (14)	*<0.001*
					*b, c, d, e*
Furosemide/loop diuretics n (%)	27 (27)	39.6 (40)	36 (35.6)	50 (50)	*0.009*
					*c*
Loop diuretics **with** simultaneous ACE inhibitor/ARB treatment n (%)	6 (6)	15 (14.9)	13 (12.9)	20 (20)	*0.034*
					*c*

Significant difference between BNP quartiles 1–4, is presented as:

1 and 2: a.

1 and 3: b.

1 and 4: c.

2 and 3: d.

2 and 4: e.

3 and 4: f.

### One-year mortality

One-year mortality rate for the study population was 34.2%. One-year mortality rate in the group with diagnosed HF was significantly higher compared to the non-HF patients (52.9% vs. 31.1%, respectively, p = 0.02). When dividing the groups into gender, the mortality was still higher in the HF group compared to the non HF group, and the difference was also significant when divided into age strata (Table 5).

**Table 5:** One year mortality

	**No HF (n = 363)**	**HF (n = 66)**	**p value (chi-square)**
Women (n = 305)	31.8%	52.3%	*0.004*
Men (n = 124)	29.4%	54.3%	*<0.001*
Total (n = 429)	31.1%	52.9%	*0.002*
**Age (years)**			
65-80 (n = 106)	16.8%	85.9%	*<0.001*
81-90 (n = 216)	27.7%	48.4%	*0.004*
90-101 (n = 107)	58.1%	78.6%	*0.001*

Patients without HF compared to patients with HF.

When comparing the groups with HF diagnosis and no HF diagnosis but with BNP > 100 ng/L, the mortality was similar (46.2% vs. 52.8%, p = 0.29).

## Discussion

The point prevalence of patients with diagnosed HF in their patient record at baseline was 15.4% but, according to BNP levels, the prevalence might have been closer to 50% if further examinations had been conducted in subjects with BNP values above the suggested cut-off for HF.

It is possible that HF diagnosis is neglected in patients with cognitive impairment as the patients without HF diagnosis tend to have a lower result on MMSE.

This study is unique in that the population studied is old and frail, with a mean age of 85 years. In most other studies of HF, this group is excluded [27]. Over 400 patients were studied, which provided substantial data for mapping the study population. Since the patients were living in nursing homes, the reports of medications and compliance with medications may have been more reliable than in other settings.

A literature review from 2010 showed a HF prevalence of 20% in elderly patients living in nursing homes [9]. The prevalence of HF was determined in five studies and the range was 15-20% [28-31], with the exception of one study that showed a prevalence of 45% [32]. In this last study, HF was diagnosed after a thorough examination by a clinical geriatrician, while information from medical records was used to confirm the diagnosis of HF in the other four studies [9,28-31]. This suggests that there are patients with undiagnosed HF that is clinically evident when the patients are more thoroughly examined.

More thorough examinations and echocardiography were shown to give HF patients a more accurate diagnosis. In a recently published study from the UK [33], the presence of HF was determined by evaluation of symptoms and signs, functional capacity, and quality of life, portable on-site echocardiography, and evaluation of medical records. The point prevalence of HF in this study was 22.8%, and additional results showed that most cases of HF were previously undiagnosed. Moreover, three-quarters of previously recorded cases were not confirmed.

An on-going Dutch study is investigating the prevalence of HF in nursing home residents after a focused clinical assessment by a geriatrician [34]. This will provide valuable information concerning undiagnosed HF, but there might be limitations to the study, such as refusal of eligible patients to participate due to the extensive examinations involved.

Our prevalence of HF diagnoses of around 15% is consistent with several other studies [4,9,34]. However, in our study about half of the study population had a BNP value above the cut-off value for HF. In addition, the prevalence of HF increases with age and one might expect that the prevalence in this patient sample would be higher than 15%.

Because of the great cost and hospitalization burden of HF, screening tests for left ventricular dysfunction among high-risk subjects (such as frail elderly persons) are required to enable implementation of intervention protocols [11]. With the suggested cut-off value for BNP, the negative predictive value is high and the BNP value should be used to exclude patients from further examination [20]. Many studies have shown the role of BNP as a predictor of morbidity [35,36]. This was also the case in this study, where the frailty (low BMI, low blood pressure, low eGFR) of patients increased with BNP levels (Table 2). Studies underline the confounders of age, gender and an impaired renal function when using the BNP value for elderly patients with HF [20,37]. Although in our study, the subjects with BNP values > 100 ng/L without HF diagnosis did not differ in gender, age nor renal function when compared to the subjects with HF diagnosis. This could emphasize the need of further screening of the patients with BNP values > 100 ng/L with echocardiography to avoid neglected HF.

Cognitive impairment is a common consequence of HF [38] and is known to be an independent prognostic marker for HF outcome since it affects quality of life, morbidity and mortality [39]. In our study, the MMSE scores were not significantly different between the first and the fourth BNP quartile. The individuals with HF diagnoses had higher scores in the MMSE test compared to patients without HF diagnoses. The patients with low MMSE scores may not have been able to express their symptoms of HF, which could have been one reason why possible HF diagnoses could have been disregarded.

If correctly diagnosed, HF is a condition in which pharmacological treatment can increase quality of life and decrease morbidity [40]. Even though most clinical trials have included younger persons, the recommendations for medical treatment of elderly patients with HF do not differ from the general recommendations. Special attention should be paid to co-morbidities, the risk of polypharmacy and renal function [40]. In this study population, renal function was not an obstacle for treatment in many patients as the mean eGFR was around 40 ml/min/1.73 m^2^.

It is important for HF patients to receive the recommended treatment for their condition. In this study, the probability of adequate medication increased if the correct diagnosis was in the patient record.

The guidelines recommend ACE inhibitors/ARBs as the first-line HF treatment, with the use of diuretics as a complement when there is volume overload [40]. The proportion of patients receiving ACE inhibitors/ARBs should therefore ideally have been higher in our study population, especially considering how common the use of loop diuretics was. The use of loop diuretics may activate the renin-angiotensin-aldosterone system [41], which plays an important role in the progression of HF, and it may also lower the GFR by decreasing the intravascular volume. It is also known to cause electrolyte imbalances [40]. Therefore, routine use of diuretics without signs of fluid retention should be avoided and has even been shown to be associated with worse outcomes in patients with HF [42].

The mortality of the patients with diagnosed HF was higher than in the non HF group. This underlines that HF is important to take into concern in the elderly. The mortality in the group with BNP >100 ng/L was similar to the group with HF which could indicate that there are patients with unrecognised HF in this group.

### Limitations of the paper

The study has limitations in that the sample used was not randomly selected, but rather selected for reasons of convenience from three different areas in Sweden, with persons living in nursing homes whose staffs was interested in joining the project being asked to participate. The nursing homes included did not differ notably from other nursing homes in Sweden [17]. There is limited access to nursing homes in Sweden and consequently the population in nursing homes reflects the group of elderly individuals in the society in greatest need of care [16]. Therefore, the results cannot be generalized to the overall population in this age group. Another limitation of the study was the unavailability of the results of echocardiography and electrocardiography, which would have provided valuable information about subjects with HF.

## Conclusions

Our study suggests that the estimated prevalence of HF in nursing homes in Sweden would be higher if BNP measurements were used to select patients for further investigation. The HF diagnosis in subjects with cognitive impairment may in some cases be neglected. The use of medications in the patients with HF diagnoses was not in accordance with current guidelines regarding use of HF medications in elderly individuals.

## Competing interests

The authors declare that they have no competing interests.

## Authors’ contributions

BBB performed the data analysis and drafted the manuscript. CJÖ, SMÖ and PM participated in the design of the study and revised the manuscript. All authors read and approved the final version of the manuscript.

## Pre-publication history

The pre-publication history for this paper can be accessed here:

http://www.biomedcentral.com/1471-2318/13/118/prepub
